# Serum Adipokines and Adipose Tissue Distribution in Rheumatoid Arthritis and Ankylosing Spondylitis. A Comparative Study

**DOI:** 10.3389/fimmu.2013.00453

**Published:** 2013-12-13

**Authors:** Éric Toussirot, Émilie Grandclément, Béatrice Gaugler, Fabrice Michel, Daniel Wendling, Philippe Saas, Gilles Dumoulin, CBT-506

**Affiliations:** ^1^University Hospital of Besançon, Clinical Investigation Center for Biotherapy INSERM CBT-506, FHU INCREASE, Besançon, France; ^2^Department of Rheumatology, University Hospital of Besançon, Besançon, France; ^3^Department of Therapeutics, University of Franche Comté, Besançon, France; ^4^University of Franche Comté, UPRES EA 4266 «Pathogens and Inflammation» SFR FED 4234, Besançon, France; ^5^LabEX LipSTIC, ANR-11-LABX-0021, Besançon, France; ^6^University Hospital of Besançon, Endocrine and Metabolic Biochemistry, Besançon, France; ^7^University of Franche Comté, UPRES EA 3920 “Cardiovascular Pathophysiology and Prevention”, SFR FED 4234, Besançon, France; ^8^UMR1098, INSERM, Besançon, France; ^9^Etablissement Français du Sang Bourgogne Franche-Comté, UMR1098, Besançon, France; ^10^Université de Franche Comté, UMR1098, Besançon, France; ^11^Department of Physical Medicine and Rehabilitation, University Hospital of Besançon, Besançon, France

**Keywords:** fat mass, body composition, visceral fat, leptin, adiponectin, cardiovascular risk

## Abstract

Rheumatoid arthritis (RA) and ankylosing spondylitis (AS) are inflammatory rheumatic diseases that may modify body composition. Adipose tissue has the ability to release a wide range of products involved in physiologic functions, but also in various pathological processes, including the inflammatory/immune response. RA and AS are both associated with the development of cardiovascular complications. It is has been established that central/abdominal, and particularly intra-abdominal or visceral adiposity is closely linked to cardiovascular events. Thus, in this study, we aimed to evaluate the body composition of patients with RA or AS compared to healthy controls (HC), with a special emphasis on the visceral region. In parallel, we measured adipose products or adipokines, namely leptin, adiponectin and its high molecular weight (HMW) isoform, resistin, and ghrelin, a gastric peptide that plays a role in energetic balance. The homeostasis model assessment for insulin resistance (HOMA-IR) and atherogenic index were used to evaluate cardiovascular risk. One hundred and twelve subjects were enrolled (30 patients with RA, 31 with AS, and 51 HC). Body composition was measured using dual-energy X-ray absorptiometry to determine total fat mass and lean mass, adiposity, fat in the android and gynoid regions, and visceral fat. Patients and HC did not differ in terms of body mass index. On the contrary, adiposity was increased in RA (*p* = 0.01) while visceral fat was also increased, but only in women (*p* = 0.01). Patients with AS tended to have lower total fat mass (*p* = 0.07) and higher lean mass compared to HC (*p* = 0.07). Leptin and leptin/fat mass were decreased in male patients with AS (*p* < 0.01), while total adiponectin and the ratio of HMW to total adiponectin were both increased in RA (*p* < 0.01). There were no changes in serum resistin and ghrelin in any group of patients. HOMA-IR and the atherogenic index were not modified in RA and AS. These results confirm that body composition was altered in RA and AS, affecting distinct soft tissue compartments. The effect of the increased visceral adipose tissue on cardiovascular risk is presumably attenuated by the favorable cardiometabolic profile in women with RA, as suggested by the normal HOMA-IR and atherogenic index.

## Introduction

Rheumatoid arthritis (RA) and ankylosing spondylitis (AS) are inflammatory joint diseases that are characterized by peripheral symmetrical polysynovitis and axial skeleton involvement, respectively. They are both associated with changes in body composition. Indeed, loss of skeletal muscle mass and accumulation of body fat are observed in patients with RA and this phenomenon is known as rheumatoid cachexia (1). In AS, a predominant loss of appendicular lean mass is described, while body fat remains unaltered (2, 3). Cachexia can be defined as a loss of skeletal muscle or lean mass and is associated with decreased muscle function, weakness and physical inactivity, and thus may strongly contribute to the disability that complicates these diseases (1). It is now well established that pro-inflammatory cytokines, such as TNF-α, play a major role in driving inflammation in RA and AS. Together with the disease activity, it is believed that such pro-inflammatory cytokines are linked to the development of body composition alterations in RA and AS (4).

It is now well recognized that RA and AS are associated with accelerated atherosclerosis, and thus, with increased cardiovascular morbidity and mortality (5). Body composition may be evaluated using dual-energy X-ray absorptiometry (DXA) and measurements include total lean and fat mass, but specific anatomical regions may also be investigated. In fact, when evaluating cardiovascular risk in patients with chronic inflammatory conditions, android fat deposits in the abdominal region are of particular interest (6, 7). Fat accumulation in the android region refers to abdominal fat that can be subclassified into subcutaneous and visceral fat (8). It has previously been demonstrated that visceral fat, rather than subcutaneous fat, is a major predictor of adverse cardiovascular events (9). Therefore, the assessment of android or visceral fat in chronic inflammatory rheumatic diseases, such as RA and AS, is a relevant issue.

In parallel, it is established that adipose tissue is not only a storage organ, but is able to produce a wide variety of factors called adipokines, such as leptin, adiponectin, resistin, and visfatin (10). These bioactive substances are involved in physiologic functions and could participate in various pathological processes, including inflammation and immunity (10–12). They also contribute to the pathophysiology of obesity and/or immune-related diseases, such as RA or AS (13). Leptin, the first adipokine to be described, is involved in appetite regulation and promotes pro-inflammatory responses (12). Adiponectin plays a central metabolic role by its insulin sensitivity and anti-atherogenic properties (14). On the other hand, the influence of adiponectin on immune response is still controversial, with the description of both pro- and anti-inflammatory effects (12, 15). Different molecular forms of adiponectin are described, with opposite effects on inflammatory processes, with the high molecular weight (HMW) form driving pro-inflammatory effects, while the low molecular form displays anti-inflammatory properties (16). Resistin is another adipokine considered to have pro-inflammatory effects (11, 12). In addition to these adipose-derived substances, ghrelin is a gastric peptide that stimulates food intake. In parallel, ghrelin has anti-inflammatory activities, especially in inflammatory joint conditions (17).

The assessment of adipose tissue and the substances it secretes is therefore of prime concern in RA and AS. Circulating adipokine levels have been widely evaluated in RA, and to a lesser extent in AS, showing elevated serum concentrations, suggesting that these adipose products contribute to the inflammatory response (11, 12). A limited number of studies have evaluated body composition in RA and AS, without specific assessment of fat distribution in the android or visceral regions (18). Since both RA and AS are associated with increased cardiovascular risk, and display changes in body composition measurements as well as altered circulating adipokine levels, we aimed to evaluate adipose tissue and its distribution in this study, with a special emphasis on the android and visceral regions, and to measure serum adipokines and ghrelin. Therefore, body composition measurements and adipokines that are linked to cardiovascular events, as well as cardiometabolic parameters such as insulin sensitivity were specifically evaluated.

## Patients and Methods

### Patients

This was a single-center, cross-sectional study. Consecutive inpatients or outpatients with RA or AS treated in the department of Rheumatology of the University Hospital of Besançon were included. Two physicians (ET, DW) recruited patients during scheduled consultations or hospitalizations. Patients with RA who fulfilled the 1987 American College of Rheumatology criteria (19) and patients with AS who met the modified New York criteria (20) were included. In the RA group, patients were receiving traditional disease-modifying anti-rheumatic drugs (DMARDs) [methotrexate, leflunomide, sulfasalazine (SLZ), or hydroxychloroquine] and/or low dose corticosteroids (CTC) (≤10 mg prednisone daily). In the AS group, patients were receiving non-steroidal anti-inflammatory drugs (NSAIDs) and/or SLZ. No patient was receiving, or had previously received a biologic agent (anti-TNF-α, abatacept, tocilizumab, rituximab, or anakinra). We excluded patients with diabetes mellitus and other endocrine disorders, as well as patients receiving high dose CTC (>10 mg prednisone daily). No patient in this study had lipid lowering drugs.

### Healthy controls

The control group consisted of healthy subjects recruited among the hospital staff, without inflammatory conditions or treatment (including CTC and lipid lowering drugs). Exclusion criteria were the same as for the patient group, i.e., subjects with diabetes and endocrine disorders were excluded.

All patients and healthy control (HC) gave written informed consent. The study protocol was performed in accordance with the Helsinki declaration and approved by the local ethics committee (*Comité d’Éthique Clinique du CHU de Besançon*).

### Methods

#### Clinical data

Clinical characteristics were recorded in each patient group. In the RA group, disease activity and functional impairment were evaluated using the Disease Activity Score (DAS28) and the Health Assessment Questionnaire (HAQ), respectively. For patients with AS, clinical activity was evaluated using the Bath Ankylosing Spondylitis Disease Activity Index (BASDAI) and the Bath Ankylosing Spondylitis Functional Index (BASFI). Weight and body mass index (BMI) were evaluated. According to their BMI, patients were defined as underweight (BMI <18.5 kg/m^2^), normal weight (18.5–24.9 kg/m^2^), overweight (25–29.9 kg/m^2^), or obese (≥30 kg/m^2^).

#### Inflammatory and immunological parameters

Erythrocyte sedimentation rate (ESR), C-reactive protein (CRP), and IL-6 serum levels [IL-6 enzyme-linked immunosorbent assay (ELISA) kit II BD OptEIA, BD Biosciences, Le Pont de Claix, France] were used to assess inflammation. Positivity for rheumatoid factors, anti-CCP antibodies and HLA-B27 status was also recorded.

#### Adipokines, ghrelin, and cardiometabolic parameters

After overnight fasting, venous blood samples were collected and centrifuged (for 10 min at 1500 *g*) as soon as possible. Serum aliquots were stored at −80°C until biochemistry assays. Total adiponectin, total ghrelin and leptin were determined by radioimmunoassay (RIA) (Millipore, St Charles, MO, USA for total adiponectin and ghrelin and Mediagnost GmbH, Reutlingen, Germany for leptin). The inter-assay coefficients of variation (CV) were 6.9, 7.9, and 7.6% for total adiponectin, ghrelin, and leptin, respectively. Resistin (R&D systems Europe Ltd., Lille, France) and HMW adiponectin (Millipore, St Charles, MO, USA) were measured by quantitative sandwich ELISA and inter-assay CVs were 7.7 and 7.8%, respectively. The ratio of HMW to total adiponectin was calculated. The lowest level that could be detected by each assay (sensitivity) was 0.5 ng/mL for leptin, 1 μg/mL for total adiponectin, 0.5 μg/mL for HMW adiponectin, 0.02 ng/mL for resistin, and 93 pg/mL for ghrelin. Glucose levels were assessed on Dimension RXL, using hexokinase-glucose-6-phosphate dehydrogenase method (Siemens Healthcare Diagnostics Ltd., Camberley, UK) with an inter-assay CV of 1.4%. Insulin was measured by an enzyme immunoassay, with an inter-assay CV of 4.6%, on an AIA 1800 (Tosoh Bioscience, Tessenderlo, Belgium). The homeostasis model assessment for insulin resistance (HOMA-IR), calculated as fasting insulin (mU/L) × fasting glucose (mmol/L)/22.5, was used to estimate insulin resistance (21). Since leptin levels are strongly associated with the amount of adipose tissue (10), we determined leptin corrected for fat mass. In addition, it is known that women have higher serum leptin compared to men (10) and a similar but more limited gender effect is also reported for adiponectin. Thus, results for leptin and adiponectin were given separately for men and women. The influence of gender on the production of other adipokines is not clearly determined and thus, resistin and ghrelin results were also given according to gender. Fasting serum total cholesterol, as well as low density lipoprotein (LDL) and high density lipoprotein (HDL) cholesterol were also evaluated. The atherogenic index, defined as the ratio of total/HDL cholesterol, was calculated.

#### Assessment of adipose tissue

A total body scan was performed using a Lunar iDXA densitometer (GE Healthcare, Madison, WI, USA). Quality control scans were performed daily during the study period. Subjects were scanned using standard imaging and positioning protocols according to the manufacturer’s instructions. Body composition was studied from the total body scan, with measurements of fat mass and lean mass. Total and regional body fat mass and lean mass were also determined. Adiposity (% fat) was defined as the ratio of total fat tissue to (total lean mass + total fat tissue). Fat distribution was evaluated as the relative proportion of fat tissue in the android (abdominal) and gynoid (hip and thigh) regions. For the measurement of android fat, a region of interest was automatically defined (from the top of the iliac crest to 20% of the distance from the top of the iliac crest to the base of the skull). Visceral adipose tissue was calculated using Lunar CoreScan software (GE Healthcare, Madison, WI, USA), which estimates specific intra-abdominal adipose tissue, excluding subcutaneous abdominal fat (8). The CV for adiposity, fat mass, and lean mass were 0.63, 0.59, and 0.45%, respectively (22).

## Statistical Analysis

Results are expressed as mean ± standard error of the mean (SEM). Due to a lack of normal distribution of the assessed variables, non-parametric tests were used. Statistical analysis between the three groups (RA, AS, and HC) involved non-parametric analysis of variance (ANOVA) using the Kruskal–Wallis test. This test was used to compare age, BMI, ESR, CRP, and IL-6, serum adipokine (serum leptin, leptin/fat mass, total and HMW adiponectin, HMW/total adiponectin ratio, resistin) and ghrelin levels, cardiometabolic parameters (fasting glycemia, insulin, HOMA-IR, total cholesterol, LDL and HDL cholesterol, total/HDL cholesterol), and body composition measurements (total fat and lean masses, adiposity, android and gynoid fat, visceral fat) between the three groups. When analysis between the three studied groups was significant, pairwise tests were performed (RA vs. HC or AS vs. HC) using the Mann–Whitney test. Qualitative data (gender) were analyzed using the Chi-square test. Spearman’s *r* was used to calculate correlations between markers of disease activity (ESR, CRP, IL-6, and DAS28 or BASDAI) and serum adipokine and ghrelin levels or body composition measurements. A *p* value < 0.05 was considered statistically significant for all tests. All analyses were performed using SigmaStat software (SSS Inc., Chicago, IL, USA).

## Results

### Clinical characteristics of the study population

The demographic and clinical characteristics of the patients and HC are shown in Table 1. A total of 112 subjects (30 patients with RA, 31 with AS, and 51 HC) were included over a 1-year period. In RA, most of the patients had traditional DMARDs and CTC (mean dose: 5.9 ± 2.8 mg daily). In patients with AS, treatments given were NSAIDs and only a limited number had SLZ or CTC (mean dose: 8.4 ± 2.8 mg daily). All patients with RA had active disease (as defined by a DAS28 ≥ 3.2), as had patients with AS (defined as a BASDAI ≥ 4). Patients with RA were older compared to patients with AS or HC (Kruskal–Wallis test: *p* = 0.0002; Mann–Whitney test RA vs. HC: *p* = 0.0005; mean difference around 10 years, range 37 to 73 in RA and 26 to 71 in HC). As expected, most enrolled patients with AS were men (93%) (Chi-square test RA vs. AS vs. HC *p* < 0.0001; Chi-square test AS vs. HC: *p* = 0.007). ESR, CRP, and IL-6 levels were elevated in RA and AS compared to HC (Kruskal–Wallis tests: all *p* < 0.0001; Mann–Whitney tests RA vs. HC and AS vs. HC, all *p* < 0.005).

**Table 1:** **Clinical and demographic characteristics of patients with rheumatoid arthritis (RA), ankylosing spondylitis (AS), and healthy controls (HC)**.

	RA	AS	HC	p*	p***
*N*	30	31	51		
Age (years) (range)	56.9 ± 1.8 (37–73)	43.8 ± 2.4 (20–65)	46.6 ± 1.5 (26–71)	0.0002	RA vs. HC: *p* = 0.0005
					AS vs. HC *p* = NS
Sex (M/F)	11/19	28/3	29/22	** <0.0001	**RA vs. HC *p* = NS
					**AS vs. HC *p* = 0.007
Body mass index (kg/m^2^) M + F	25.5 ± 0.8	24.1 ± 0.6	24.9 ± 0.7	NS	
Body mass index (kg/m^2^) M	25.7 ± 1.2	24.3 ± 0.7	24.8 ± 0.6	NS	
Body mass index (kg/m^2^) F	25.3 ± 1	22 ± 1.3	25 ± 1.3	NS	
Underweight (*N*)	1	1	1		
Normal weight (*N*)	14	19	27		
Overweight (*N*)	10	9	19		
Obese (*N*)	5	2	4		
Disease duration (years)	11.7 ± 1.6	13.1 ± 1.9			
Treatments	MTX *N* = 23	NSAIDs *N* = 23			
	LFM *N* = 4	SLZ *N* = 4			
	SLZ *N* = 2	CTC *N* = 3			
	CTC *N* = 26				
Rheumatoid factors (%)	83.3				
Anti CPP (%)	56.6				
HLA-B27 (%)		83.4			
DAS28	3.5 ± 0.2				
HAQ	1.1 ± 0.1				
BASDAI		4.4 ± 0.3			
BASFI		4.1 ± 0.5			
ESR (mm/h)	21.8 ± 3.1	28.5 ± 4.3	12 ± 3.2	<0.0001	RA vs. HC *p* < 0.0001
					AS vs. HC *p* = 0.0002
CRP (mg/L)	12.8 ± 2.8	29.5 ± 6.1	4.4 ± 1.1	<0.0001	RA vs. HC *p* < 0.0001
					AS vs. HC *p* < 0.001
IL-6 (pg/mL)	34.1 ± 12.3	19.4 ± 3.4	14.4 ± 7.7	<0.0001	RA vs. HC *p* < 0.0001
					AS vs. HC *p* < 0.0001

(M, male; F, female; DAS28, disease activity score 28 joints; HAQ, health assessment questionnaire; BASDAI, Bath ankylosing spondylitis disease activity index; BASFI, Bath ankylosing spondylitis functional index; MTX, methotrexate; LFM, leflunomide; SLZ, sulfasalazine; HCQ, hydroxychloroquine; CTC, corticosteroids; ESR, erythrocyte sedimentation rate; CRP, C-reactive protein; results were given as mean ± SEM; *Kruskal–Wallis test; **Chi-square test; ***Mann–Whitney test).

#### Serum adipokine levels and cardiometabolic parameters

No significant differences were observed between groups for fasting glycemia, HOMA-IR, LDL cholesterol, total/HDL cholesterol, resistin, and ghrelin levels (Table 2). Insulin levels differed significantly between the three groups (Kruskall Wallis test *p* = 0.01) and this difference was related to lower levels in the AS group (Mann–Whitney test AS vs. HC: *p* = 0.009). Total cholesterol and HDL cholesterol also differed significantly (Kruskal–Wallis tests *p* = 0.0008 for both) due to increased HDL levels in the RA group (Mann–Whitney test RA vs. HC: *p* = 0.005) and decreased total cholesterol and HDL levels in the AS group (Mann–Whitney tests *p* = 0.002 and 0.05, respectively). However, the atherogenic index of total/HDL cholesterol was found to be similar between the three groups. The three groups differed in terms of serum leptin levels (Kruskal–Wallis test *p* < 0.0001), but this difference reflected the gender distribution (as stated above, there was a high proportion of men in the AS group). Thus, leptin results were analyzed in men and women separately. No difference in serum leptin and leptin/fat mass ratio was found between the three groups of women. Conversely, leptin and leptin/fat mass differed significantly between groups in men (Kruskal–Wallis test *p* = 0.03 and 0.01, respectively). Pairwise tests showed that leptin and leptin/fat mass were significantly decreased in male AS patients (Mann–Whitney tests AS vs. HC *p* = 0.009 and 0.003, respectively), while the levels of leptin and the leptin/fat mass ratio were comparable between male subjects with RA and HC. Total adiponectin and the ratio of HMW/total adiponectin differed significantly between groups (Kruskal–Wallis tests *p* = 0.02 and *p* < 0.0001, respectively). These differences were driven by higher total adiponectin levels in RA compared to HC (Mann–Whitney tests RA vs. HC for total adiponectin and total/HMW adiponectin: *p* = 0.008 and *p* < 0.0001, respectively). HMW adiponectin also tended to be higher in patients with RA, but without reaching the significance level (Kruskal–Wallis test *p* = 0.09). Finally, we also examined whether there was a gender effect for resistin and ghrelin. However, no significant difference was observed in serum resistin and ghrelin levels when men and women were analyzed separately (Kruskal–Wallis test, all *p* > 0.05).

**Table 2:** Metabolic parameters, serum ghrelin and adipokine levels of patients with rheumatoid arthritis (RA), ankylosing spondylitis (AS), and healthy controls (HC) (M, male; F, female; *Kruskal–Wallis test; **Mann–Whitney test).

	RA *N* = 30	AS *N* = 31	HC *N* = 51	p*	p**
Glycemia (mmol/L)	5 ± 0.1	4.9 ± 0.1	4.6 ± 0.1	NS	
Insulin (μIU/L)	8.4 ± 0.9	5.6 ± 0.5	10.7 ± 1.6	0.01	RA vs. HC *p* = NS
					AS vs. HC *p* = 0.009
HOMA-IR	1.9 ± 0.3	1.2 ± 0.1	2.4 ± 0.4	NS	
Total cholesterol (g/L)	2.2 ± 0.1	1.8 ± 0.5	2.1 ± 0.1	0.0008	RA vs. HC *p* = NS
					AS vs. HC *p* = 0.002
LDL cholesterol (g/L)	1.2 ± 0.1	1.1 ± 0.1	1.2 ± 0.1	NS	
HDL cholesterol (g/L)	0.6 ± 0.1	0.49 ± 0.05	0.5 ± 0.1	0.0008	RA vs. HC *p* = 0.005
					AS vs. HC *p* = 0.05
Total/HDL Cholesterol	3.6 ± 0.2	3.8 ± 0.2	3.9 ± 0.2	NS	
Leptin (ng/mL) M + F	15.3 ± 2.4	3.5 ± 0.6	14.3 ± 2.3	<0.0001	RA vs. HC *p* = NS
					AS vs. HC *p* < 0.0001
Leptin M (ng/mL)	7.5 ± 3.5	2.8 ± 0.5	5.1 ± 0.7	0.03	RA vs. HC *p* = NS
					AS vs. HC: *p* = 0.009
Leptin F (ng/mL)	19.8 ± 2.7	9.1 ± 1.5	25.2 ± 4.2	NS	
Leptin/fat mass (ng/mL/g) M + F	0.54 ± 0.06	0.16 ± 0.02	0.53 ± 0.07	<0.0001	RA vs. HC *p* = NS
					AS vs. HC *p* < 0.0001
Leptin fat mass M (ng/mL/g)	0.24 ± 0.07	0.13 ± 0.01	0.26 ± 0.05	0.01	RA vs. HC *p* = NS
					AS vs. HC: *p* = 0.003
Leptin/fat mass F (ng/mL/g)	0.72 ± 0.06	0.41 ± 0.06	0.87 ± 0.12	NS	
Total adiponectin M + F (μg/mL)	13.2 ± 1.1	9.9 ± 0.9	9.4 ± 0.5	0.02	RA vs. HC *p* = 0.008
					AS vs. HC *p* = NS
Total adiponectin M (μg/mL)	12.1 ± 1.1	8.8 ± 0.7	7.9 ± 0.7	0.01	RA vs. HC *p* = 0.003
					AS vs. HC NS
Total adiponectin F (μg/mL)	13.8 ± 1.7	20 ± 3.5	11.4 ± 0.6	NS	
HMW adiponectin M + F (μg/mL)	9.4 ± 0.9	7.2 ± 0.8	7.6 ± 0.5	0.09	
HMW adiponectin M (μg/mL)	8.4 ± 1.1	6.2 ± 0.6	6.2 ± 0.6	NS	
HMW adiponectin F (μg/mL)	10 ± 1.3	16.3 ± 3.9	9.6 ± 0.5	NS	
HMW/total adiponectin M + F	69.8 ± 1.7	70.4 ± 2	80.4 ± 1.5	<0.0001	RA vs. HC *p* < 0.0001
					AS vs. HC NS
HMW/total adiponectin M	68.1 ± 3	69.4 ± 1.9	79.6 ± 2.2	0.002	RA vs. HC *p* = 0.01
					AS vs. HC *p* = 0.002
HMW/total adiponectin F	70.8 ± 2	79.5 ± 8.8	81.5 ± 1.9	0.005	RA vs. HC *p* = 0.0008
					AS vs. HC NS
Resistin M + F (ng/mL)	11.4 ± 1.1	13.5 ± 1.1	10.6 ± 0.7	NS	
Resistin M (ng/mL)	12.4 ± 2.1	13.7 ± 1.2	11.7 ± 0.9	NS	
Resistin F (ng/mL)	10.7 ± 1.3	11.4 ± 1.5	9.2 ± 0.8	NS	
Ghrelin M + F (pg/mL)	1220.2 ± 75.8	1272.6 ± 70.3	1091.9 ± 43.9	NS	
Ghrelin M (pg/mL)	1091.5 ± 106.7	1248 ± 75.1	1035.7 ± 50.8	0.065	
Ghrelin F (pg/mL)	1294.6 ± 100.5	1502.7 ± 156	1166.1 ± 75.4	NS	

#### Adipose tissue distribution

**Figure 1:**
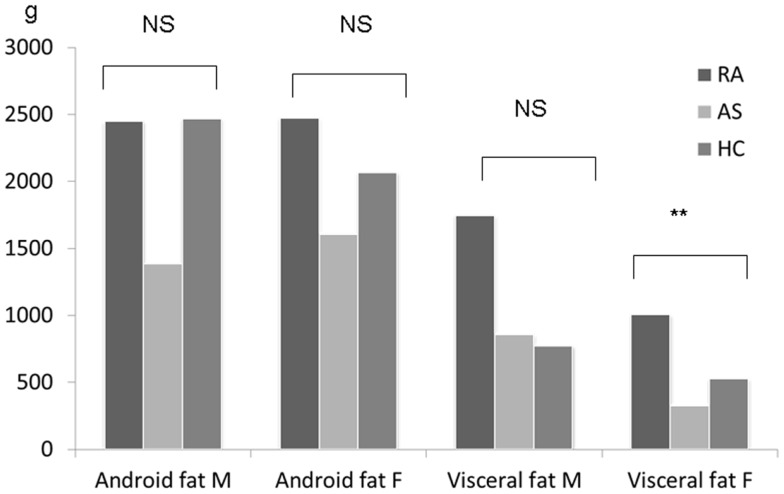
**Measurement of fat in the android and visceral regions in patients with rheumatoid arthritis (*N* = 30), ankylosing spondylitis (*N* = 31), and healthy controls (*N* = 51)**. Results are given for men and women. Comparisons were performed using the Kruskal–Wallis test; **p ≤ 0.01.

Patients and HC did not differ regarding their BMI (in the whole group, or analyzed separately in men and women) (Kruskal–Wallis tests, all *p* > 0.05). The distribution of underweight, normal weight, overweight, and obese subjects in the three groups is shown in Table 1. Since the proportion of male patients was higher in the AS group, the distribution of adipose tissue was examined according to gender. Results are given first for the whole group, and then, for men and women separately. Total fat mass was significantly different between the three groups (Kruskal–Wallis test *p* = 0.01); there was no significant difference in pairwise testing between RA and HC, while there was a tendency toward decreased fat mass in AS (Mann–Whitney test *p* = 0.07) (Table 3). When examining total fat mass in men or in women separately, no significant difference was found between the three groups. Similar results were obtained for lean mass: total lean mass significantly differed between the three groups overall (Kruskal–Wallis test *p* = 0.004), explained by a tendency toward higher lean mass in the AS group (Mann–Whitney test AS vs. HC *p* = 0.07), but again, no difference was found between the three groups when men and women were analyzed separately. The three groups also differed overall regarding adiposity (Kruskal–Wallis test *p* = 0.0001) and this difference was explained by higher values in RA (Mann–Whitney test RA vs. HC *p* = 0.01) while lower values were observed in AS (Mann–Whitney test AS vs. HC *p* = 0.01). However, these differences did not persist when men and women were analyzed separately. Android fat was similar between the three groups although there was a tendency toward decreased fat mass in the AS group, without reaching the significance level (Kruskal–Wallis test *p* = 0.055). Conversely, gynoid fat was found to be significantly different (Kruskal–Wallis test *p* = 0.002), and this difference was related to lower levels in the AS group compared to HC (Mann–Whitney test *p* = 0.0003). However, this difference was not confirmed when men and women were analyzed separately. Finally, the amount of visceral fat was found to be significantly different between the three groups (Kruskal–Wallis test *p* = 0.03), a difference that was observed only in the RA group (Mann–Whitney test RA vs. HC *p* = 0.01) (Figure 1), and especially in women (Kruskal–Wallis test in women: *p* = 0.01 and Mann–Whitney test in women RA vs. HC *p* = 0.01).

**Table 3:** Body composition measurements of patients with rheumatoid arthritis (RA), ankylosing spondylitis (AS), and healthy controls (HC) (M, male; F, female; *Kruskal–Wallis test; **Mann–Whitney test).

	RA *N* = 30	AS *N* = 31	HC *N* = 51	p*	p**
Total fat mass M + F (g)	24997.8 ± 1645.5	18516.8 ± 1773.4	21639.5 ± 1357.6	0.01	RA vs. HC *p* = NS
					AS vs. HC *p* = 0.07
Total fat mass M (g)	22524 ± 3414.1	18195.8 ± 1955	20014.5 ± 1552.4	NS	
Total fat mass F (g)	26430 ± 1681.7	21513 ± 854.5	23728.8 ± 2341.8	NS	
Total lean mass M + F (g)	42270.9 ± 1520.7	49839.3 ± 1251.2	46073.3 ± 1444.2	0.004	RA vs. HC *p* = NS
					AS vs. HC *p* = 0.07
Total lean mass M (g)	50665.7 ± 1280.7	51330.8 ± 1022.6	52967.2 ± 1431.1	NS	
Total lean mass F (g)	37410.7 ± 1332.7	35918 ± 1896.2	37209.7 ± 893	NS	
Adiposity (%) M + F	36.7 ± 1.6	25.9 ± 1.6	31.9 ± 1.3	0.0001	RA vs. HC *p* = 0.01
					AS vs. HC *p* = 0.01
Adiposity M (%)	29.4 ± 2.9	24.7 ± 1.6	26.5 ± 1.3	NS	
Adiposity F (%)	40.9 ± 1.1	37.5 ± 1.8	38.8 ± 1.6	NS	
Android fat M + F (g)	2465.6 ± 226.1	1811.8 ± 236.6	2293.1 ± 223.8	0.055	RA vs. HC *p* = NS
					AS vs. HC *p* = NS
Android fat M (g)	2448.4 ± 492.6	1384.5 ± 261.6	2469.4 ± 356.7	NS	
Android fat F (g)	2475.6 ± 227.9	1607.7 ± 163.2	2066.4 ± 227.7	NS	
Gynoid fat M + F (g)	4322.6 ± 267.1	3050 ± 300.9	4601.1 ± 402.9	0.002	RA vs. HC *p* = NS
					AS vs. HC *p* = 0.0003
Gynoid fat M (g)	3828.8 ± 589.1	2877.9 ±314.9	4337 ± 663.4	NS	
Gynoid fat F (g)	4608.4 ± 239.8	4656.3 ± 334.9	4940.7 ± 355.3	NS	
Visceral fat M + F (g)	1286.8 ± 210.2	8061 ± 123.1	770.6 ± 92.9	0.03	RA vs. HC *p* = 0.01
					AS vs. HC *p* = NS
Visceral fat M (g)	1746.9 ± 482.9	857.7 ± 132.6	959.4 ± 135.1	NS	
Visceral fat F (g)	1005.7 ± 145.3	324 ± 78.6	527.8 ± 103.2	0.01	RA vs. HC *p* = 0.01

#### Relationships between adipose tissue measurements or serum adipokine levels and disease activity

No relationship was observed between leptin or leptin/fat mass and markers of disease activity in patients with AS (in the whole group or in male AS patients) (all *p* > 0.05). In patients with RA, HMW/total adiponectin correlated with the DAS28 score (*r* = −0.42, *p* = 0.02). In AS, total fat mass was correlated with CRP levels (*r* = −0.37, *p* = 0.04), while lean mass correlated with ESR (*r* = −0.38, *p* = 0.04) and weakly with IL-6 (*r* = −0.35, *p* = 0.053) and BASDAI (*r* = −0.32, *p* = 0.08). Finally, adiposity correlated with DAS28 in patients with RA (*r* = −0.43, *p* = 0.02), while there was a tendency toward a correlation with CRP in AS (*r* = −0.37, *p* = 0.07). There was no relationship between visceral fat mass and indices of disease activity in patients with RA.

## Discussion

In this study including patients and HC with comparable BMI, abnormal adipose tissue distribution was found in both RA and AS. Patients with RA were characterized by an accumulation of fat in the visceral region, especially in women, while for AS, fat mass was decreased, albeit without reaching statistical significance. In parallel, there was a tendency toward increased lean mass in AS patients. These results were correlated with disease activity, particularly adiposity in patients with RA or lean mass in patients with AS.

There are a limited number of studies evaluating body composition in RA and this study is the first to specifically measure the intra-abdominal/visceral region. Body composition has been assessed in RA in patients with early or longstanding disease. In early RA, a low lean mass was found in both men and women, while only women had higher BMI, body fat mass, and fat mass ratio (defined by the fat localized to the trunk divided by the sum of the fat of the arms and legs) than the control population (23). In patients with later disease, similar changes involving fat and lean masses were observed, but affecting both men and women (24). Fat distribution was altered with a clear shift to the abdominal region in most of these studies (23–25). The parallel loss of skeletal muscle and increase in fat makes it possible to define body composition phenotypes such as sarcopenia, overfat, and sarcopenic obesity (18). These different studies found that such phenotypes were highly represented in RA with long disease duration (24). In an Asian population from Vietnam, women with early disease also had a high proportion of similar unhealthy body composition phenotypes (26). In all these studies, body composition was assessed using DXA, which evaluated total body and regional fat mass. They showed abnormal values for all measures of adiposity, including truncal fat. This is a well known factor for cardiovascular disease in non RA patients, and it is assumed that it could have similar effects in RA (9). However, these studies of body composition in RA cannot specify whether the fat was localized subcutaneously or in the abdominal cavity. Indeed, an excess of intra-abdominal fat or visceral adipose tissue has been reported to be detrimental and associated with metabolic abnormalities including hyperinsulinemia, insulin resistance, glucose intolerance, lipid abnormalities, inflammation, and endothelial dysfunction (6, 13). Moreover, excess visceral adipose tissue has been significantly correlated with various cardiometabolic abnormalities, independently of the amount of subcutaneous fat (9). In our female patients with RA, we found an excess of visceral adipose tissue and this may influence their cardiovascular risk. A number of previous studies in RA have shown an association between abnormal body composition and CRP, but also HAQ and rheumatoid factor positivity (23, 24). Truncal fat in patients with RA was selectively associated with CRP levels (27). However, this association between markers of disease activity or severity and fat mass has not always been confirmed (25). We were unable to demonstrate such an association, especially between visceral fat and CRP and/or IL-6 levels. However, DAS28 correlated with adiposity, a finding similar to the observations in Vietnamese women with RA (26). These results suggest the influence of disease activity and thus persistent inflammation on fat deposition and distribution. Lean mass was preserved in our patients with RA, contrary to previous works (23, 24). The decrease in skeletal muscle contributes to sarcopenia, a well established feature of RA that could induce weakness, fatigue, disability, and increased risk of falls (23). Our population of RA patients did not have this body composition phenotype. Despite high disease activity and a mean disease duration of 11 years, the HAQ score was not very high in our patients. This could explain maintained physical activity and muscle function, and thus the absence of muscle loss. In addition, we previously reported that patients with RA had high growth hormone levels, and such anabolic factors may contribute to muscle mass (28). Conversely, the majority of our patients had CTC at a low dosage, a treatment that can induce loss of skeletal muscles (29).

Our group of patients with AS were characterized by a decrease in total fat mass that almost reached statistical significance, as well as a significant decrease in adiposity, while total lean mass tended to be increased. Results were less salient when this patient group was divided according to gender, and thus, results for adiposity may reflect the low proportion of female AS patients. In addition, visceral fat was comparable between AS and HC. Previous works evaluating body composition in AS using DXA are limited. A previous study by our group did not observe differences in lean and fat masses between patients and HC (3), and similar results have also been reported by others (30). However, these studies examined total body composition measurements (total fat and lean mass) and not the regional distribution of fat mass. Indeed, in patients with very long disease duration, appendicular lean mass was diminished compared to age- and sex-matched controls (2). This loss of muscle mass was significantly associated with reduced upper and lower body strength, providing evidence for clinical features of cachexia. By contrast, total fat and truncal fat were not modified in this AS population. Body composition was also assessed by methods other than DXA including bioelectrical impedance analysis (31). Body fat was found to be reduced using this method, while air-displacement plethysmography did not show any difference between AS and controls for the percentage of fat mass or fat free mass (32). The disparities in these results may be explained by the patient characteristics, the disease severity and duration, the treatment given and the methods used for assessing body composition (DXA vs. other methods). However, DXA is considered as the reference method for measurements of body composition, compared to bioelectrical impedance assessment. There was a tendency toward low fat mass and a high lean mass in our patients with AS. This may be related to preserved physical activity, as suggested by a low mean BASFI score. In addition, we observed an inverse relationship between measures of disease activity (ESR, IL-6, and BASDAI) and lean mass. Conversely, the loss of adipose tissue in our AS group could be explained by the systemic inflammation, as suggested by the relationship between fat mass and CRP levels. However, we did not specifically evaluate appendicular (arm and leg) lean mass, and thus, selective muscle loss cannot be ruled out in our AS population.

Abnormal values for certain circulating adipokines were found in our study. Resistin and ghrelin showed comparable levels between the three groups, even when men and women were analyzed separately, whereas leptin was significantly decreased in male AS patients, and total adiponectin and HMW/total adiponectin ratio were increased in patients with RA. Circulating adipokines have been extensively studied in RA (33) and to a lesser extent in AS (31, 34, 35). The interest of adipokines in inflammatory rheumatic diseases comes from the discovery of the biological properties of these proteins, and especially, their interplay with the immune system. Leptin has a globally pro-inflammatory effect by inducing a Th1 response (10, 12). In RA, serum leptin levels were found to be higher than in controls in most previous analyses, but some studies found no differences [for review, see Ref. (12)]. These discrepancies may be explained by the lack of adjustment for BMI or the absence of analysis by gender. In our study, we adjusted leptin for the total fat mass (and not the BMI), a correction that seems more relevant since leptin is a direct surrogate marker of the amount of adipose tissue (10). Leptin has previously been evaluated in AS by our group and we found similar results as compared to the present study (34). Both decreased and increased levels of leptin in AS have been reported by other studies, without any clear explanation for these contradictory results (31). Despite the lack of association between leptin and disease activity, we can hypothesize that decreased leptin levels in AS may favorably influence the inflammatory response in AS (34). Total adiponectin was increased in our RA group with a weak parallel change in HMW adiponectin. Again, adiponectin has previously been evaluated in RA showing increased levels in general (33). Adiponectin has been determined to be a factor associated with disease severity or joint destruction: two studies reported that baseline adiponectin is related to radiographic joint damage (36, 37), while the opposite was found in a third study (38). One explanation for these discrepancies is that adiponectin exists in different isoforms, with the HMW form driving inflammation (14, 16). Studies conducted to date in RA measured exclusively total adiponectin, and not its isoforms. Our results suggest that when studying adiponectin in an inflammatory condition, the assessment of its different isoforms is required.

Serum ghrelin was comparable between the three groups in our study. Ghrelin is a gastric peptide involved in energetic balance: contrary to leptin, it stimulates appetite and decreases fat utilization. We previously observed elevated ghrelin levels in AS, and this result was thought to mitigate inflammation in these patients (34). Indeed, ghrelin is able to inhibit the release of pro-inflammatory cytokines, such as IL-1, IL-6, and TNFα. We did not confirm these previous results in this study, and this may be related to the patient characteristics.

Since adipose tissue is at the crossroads of energy homeostasis, inflammation, and atherosclerosis, the metabolic profile of our patients was also evaluated. We used HOMA-IR to determine insulin sensitivity and this parameter was not affected in any group. Resistin is described as an adipokine related to insulin resistance in animal models, although the evidence of this effect in humans is less clear (13). However, our patients had resistin levels comparable to those of HC. Conversely, total adiponectin (with, in parallel, HMW adiponectin) was increased in the RA group. Besides its effect on inflammation, adiponectin is known as a protective metabolic factor. Indeed, adiponectin promotes insulin sensitivity, and thus, is decreased in patients with obesity, diabetes, or metabolic syndrome. Adiponectin is considered to be a protective cardiovascular factor, and it has been established that it can predict future cardiovascular events in patients with coronary heart disease (13). When considering the normal results of HOMA-IR and resistin levels together with the normal atherogenic index, as well as the increased adiponectin levels, the cardiometabolic profile of our patients with RA thus seems favorable.

Finally, this study has strengths and limitations. We evaluated the amount and distribution of adipose tissue, focusing for the first time on the visceral region. These measurements were performed together with the assessment of their derived products and specific cardiometabolic indexes. High molecular adiponectin was also evaluated in this study. However, a low proportion of female AS patients was evaluated and this could influence our results. For this reason, we carefully analyzed our patients according to gender. A clear sexual dimorphism is established for serum leptin, and to a lesser extent, for adiponectin, with women having higher serum leptin and adiponectin that men. For resistin, it is thought that women also have higher serum levels (39) whereas there is no influence of sex on serum ghrelin. In addition, our RA patients were older compared to AS and HC subjects. Aging could influence serum adipokines: in fact, it has been demonstrated that the levels of adiponectin (both in male and female subjects) and leptin (in males) were significantly higher in older subjects compared to their younger counterparts (40), but these results were observed in a population aged between 70 and 80 years. Our RA patients and HC had a limited age difference (around 10 years) and we did not include patients aged over 73 years. Presumably, the age difference between RA and HC did not influence our adipokine results.

## Conclusion

We conclude that body composition is altered in both RA and AS, but with different soft tissue impact. Patients with RA had increased measurements of adiposity and visceral fat deposition, especially in women, while in AS, fat mass was decreased and lean mass increased. There was no accumulation of visceral fat in AS. These differences in body composition certainly reflect distinct pathophysiological mechanisms. Patients with RA and AS have also distinct circulating adipokine profiles. Increased fat mass in the visceral region may potentially worsen the cardiovascular risk in women with RA, but the favorable cardiometabolic profile in these patients may attenuate the deleterious effect of this fat deposition. However, longitudinal studies are warranted to evaluate the real impact of visceral fat accumulation in RA on cardiovascular risk, together with the levels of circulating adipokines, such as adiponectin.

## Authors Contribution

Éric Toussirot was the main investigator: he designed the study and drafted the manuscript. He helped recruit the patients involved in the study, analyzed the results, and performed the statistical analysis. Gilles Dumoulin was responsible for assessing laboratory parameters. He helped with study design and preparation of the manuscript by writing the methods section. He analyzed and contributed to the discussion of the results. Émilie Grandclément supervised sample collection, and performed adipokine measurements. Philippe Saas contributed to the discussion of the results and critical reading of the manuscript. Béatrice Gaugler performed cytokine assessments. Daniel Wendling and Fabrice Michel contributed to the study as clinical investigators. All authors read and approved the final version of the manuscript for submission.

## Conflict of Interest Statement

The authors declare that the research was conducted in the absence of any commercial or financial relationships that could be construed as a potential conflict of interest.
